# Supplementary material for: CMG2/ANTXR2 regulates extracellular collagen VI which accumulates in hyaline fibromatosis syndrome
Source: Nat Commun. 2017 Jun 12;8:15861. doi: 10.1038/ncomms15861 (PMC5472780; doi:10.1038/ncomms15861)
Supplement: Supplementary Information [file ncomms15861-s1.pdf]

Type of file: PDF
Size of file: 0 KB
Title of file for HTML: Supplementary Information
Description: Supplementary figures, supplementary tables, supplementary note and supplementary references.

Type of file: PDF
Size of file: 0 KB
Title of file for HTML: Peer review file
Description:

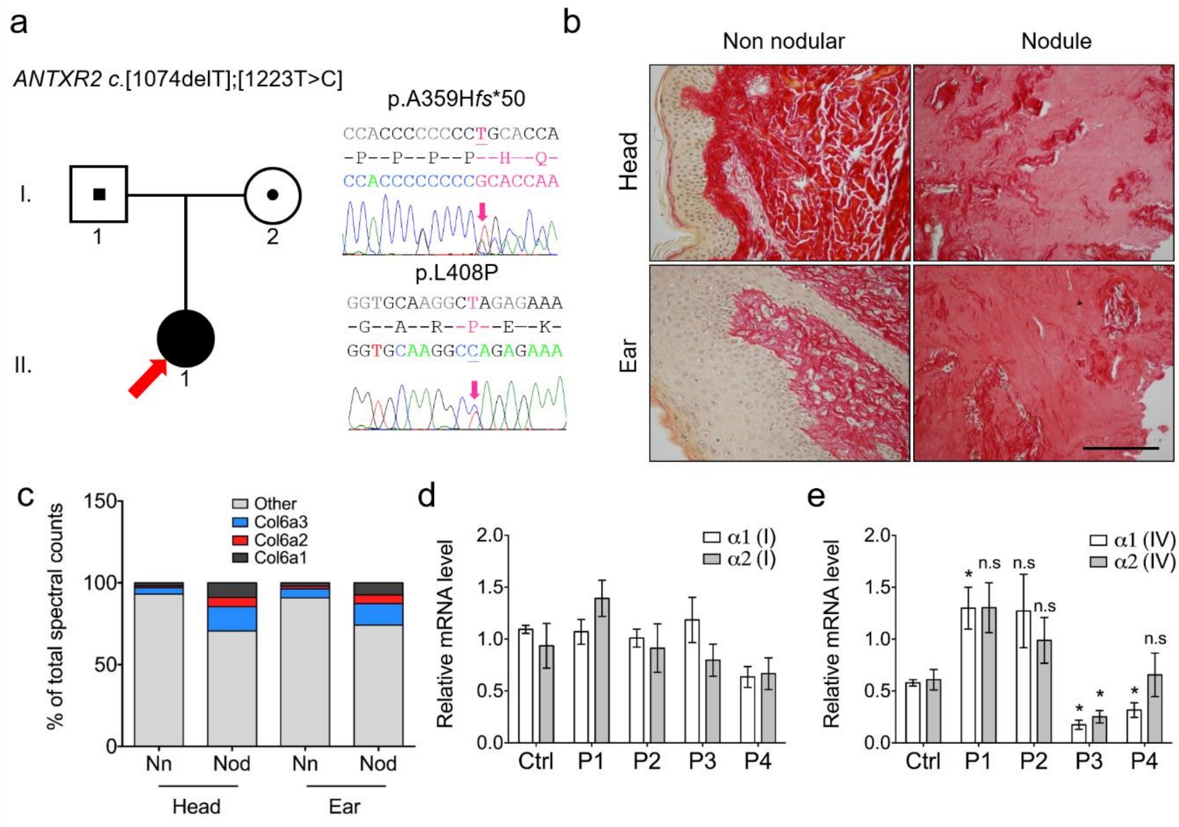

Supplementary Figure 1. (a) Abridged pedigrees of a HFS patient family. Squares represent males; circles represent females; open symbols, unaffected individuals and filled symbols, affected mutation. On the right of the pedigree is the HFS patient electropherograms with altered nucleotides marked in pink (reference sequence top and actual read bottom). Underlined nucleotides indicate deletion or missense mutation. **(b)** Sirius red staining of formalin fixed non-nodular skin and nodule from the ear and the head of the HFS patient. Scale bar, 200µm. **(c)** Proportion (in %) of the α1(VI), α2(VI), and α3(VI) peptides detected by MS in homogenate from non-nodular skin (Nn) and nodules (Nod) in the head and ear of HFS patient, compared to the total number of peptides detected in the same samples. **(d-e)** Quantitative RT-PCR analysis of mRNAs coding for the α1 and α2 chains of collagen I (left) and collagen IV (right) in fibroblast cultures from unaffected control and four HFS patients (P1-P4) (Error bars represent s.e.m; n = 3; *, $p < 0.05$, two-tailed unpaired t-test compared to control; n.s., not significant).

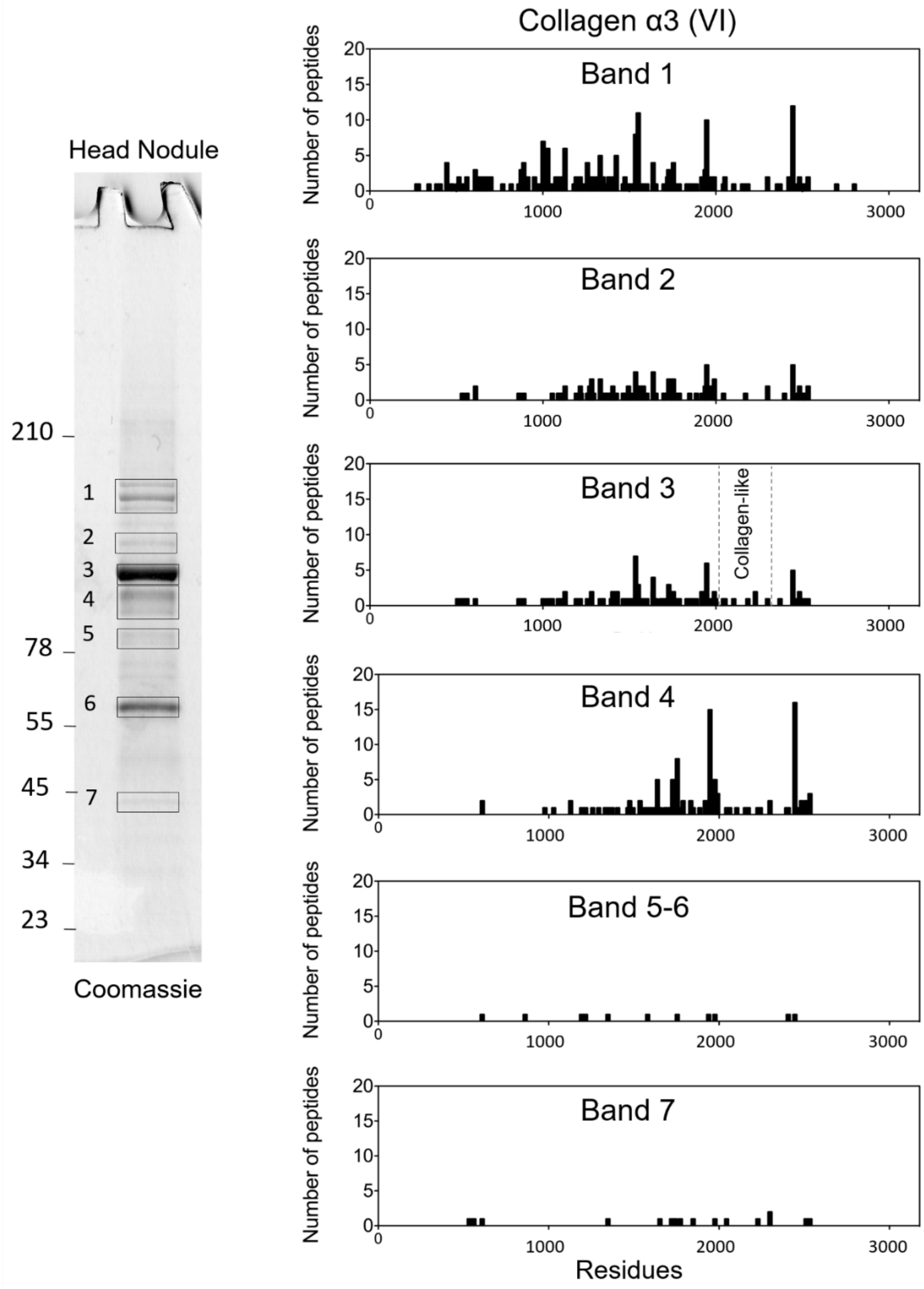

Supplementary Figure 2. Multiple band of different molecular weight were excised and the protein content analyzed by mass-spectrometry. For each band, the number and position of each collagen α3 (VI) peptides detected by mass-spectrometry are mapped on the complete sequence of the collagen α3 (VI) protein.

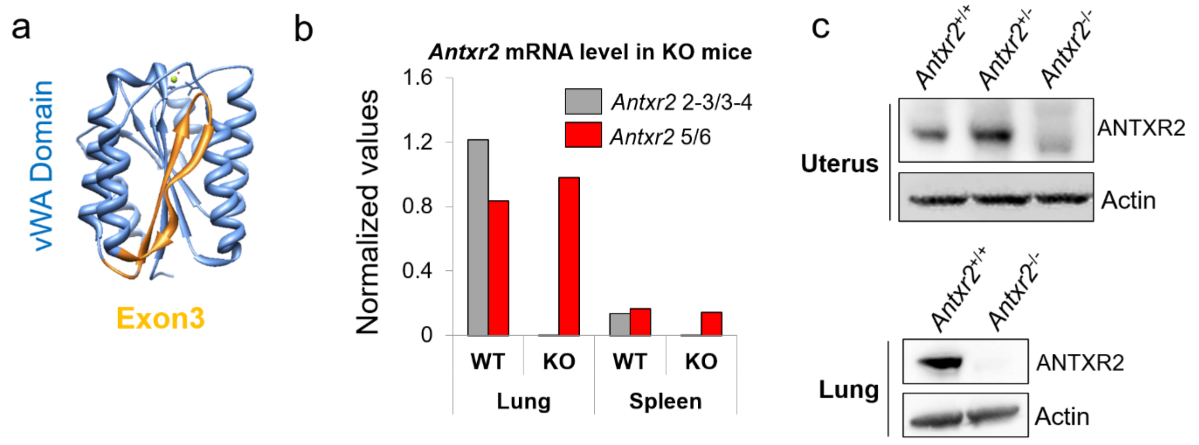

Supplementary figure 3: Generation of *Antxr2*$^{-/-}$ mice. (a) Schematic representation of CMG2 vWF-A domain based on the crystal structure (PDB ID: 1SHT)[1]. The image was realized with Chimera. A targeted deletion of exon 3 of the *Antxr2* gene was used as a strategy to generate *Antxr2*$^{-/-}$ mice. **(b)** CMG2 mRNA expression was determined by quantitative RT-PCR in lung and spleen of wild-type (WT) and *Antxr2*$^{-/-}$ (KO) mice. To confirm the correct deletion of exon 3, two sets of primers were used, set spanning exons 2-3 and 3-4, and the other one spanning exons 5-6. **(c)** Tissue lysates from uterus and lung of *Antxr2*$^{+/+}$, *Antxr2*$^{+/-}$ and *Antxr2*$^{-/-}$ littermate mice were analyzed by SDS-PAGE and western blotting for mouse ANTXR2 using 4-12% Bis-Tris gradient gels under reducing condition. Actin was used as a loading control.

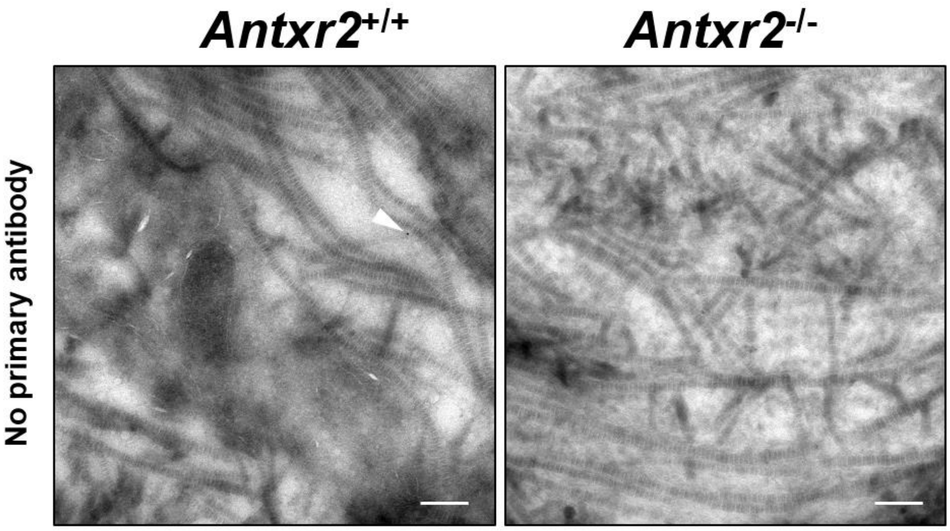

Supplementary figure 4: Control staining of Collagen VI immunogold labeling of *Antxr2*$^{+/+}$ and *Antxr2*$^{-/-}$ uterine myometrial layer analyzed by transmission electron microscopy. The white arrowhead point to a single gold particle**.** Scale bar = 200 nm.

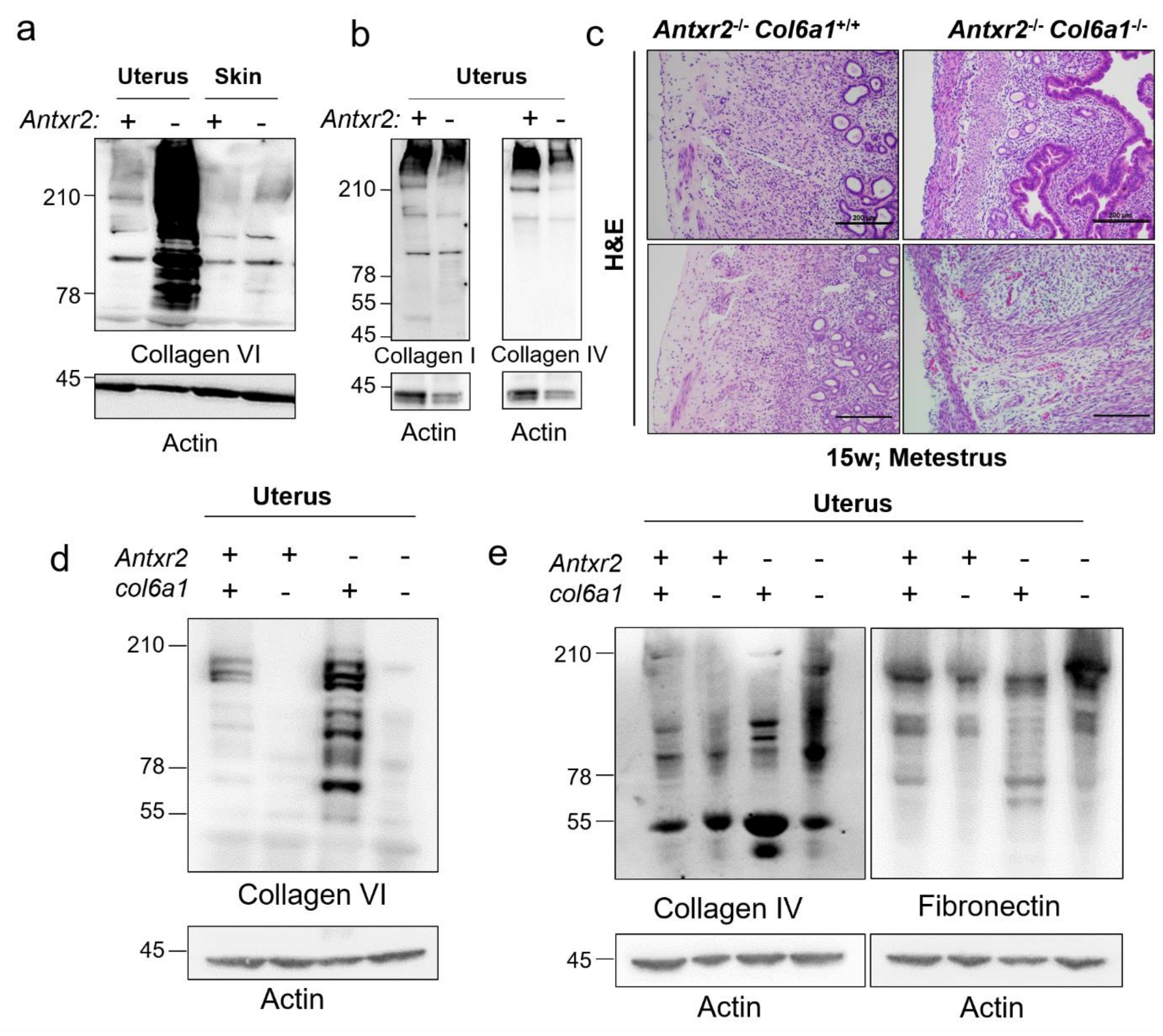

Supplementary Figure 5. (a, b) Uterus and skin tissue lysates (40µg) from *Antxr2*$^{+/+}$ and *Antxr*$^{-/-}$ mice were analyzed by SDS-PAGE using 4-12% Bis-Tris gradient gels under reducing condition and western blotting against collagen VI (a), collagen I and collagen IV (b). Actin is used as a loading control. Migration of the molecular weight markers (in kDa) is indicated on the left. A severe, diffuse and band-like accumulation of collagen VI is observed in the uterus of *Antxr2*$^{-/-}$ mice. Representative western blots of at least n = 3 mice. **(c)** Haematoxylin-eosin staining of uterine tissues from two different 15-week-old *Antxr2*$^{+/+}$::*Col6a1*$^{+/+}$, *Antxr2*$^{-/-}$::*Col6a1*$^{-/-}$ mice in metestrus. Scale bar, 200µm. **(d, e)** Uterus lysates from *Antxr2*$^{+/+}$::*Col6a1*$^{+/+}$, *Antxr2*$^{+/+}$::*Col6a1*$^{-/-}$, *Antxr2*$^{-/-}$::*Col6a1*$^{+/+}$, and *Antxr2*$^{-/-}$::*Col6a1*$^{-/-}$ mice were analyzed by SDS-PAGE using 4-12% Bis-Tris gradient gels under reducing condition and western blotting for collagen VI (c) or for collagen IV and fibronectin (d). Migration of the molecular weight markers (in kDa) is indicated on the left.

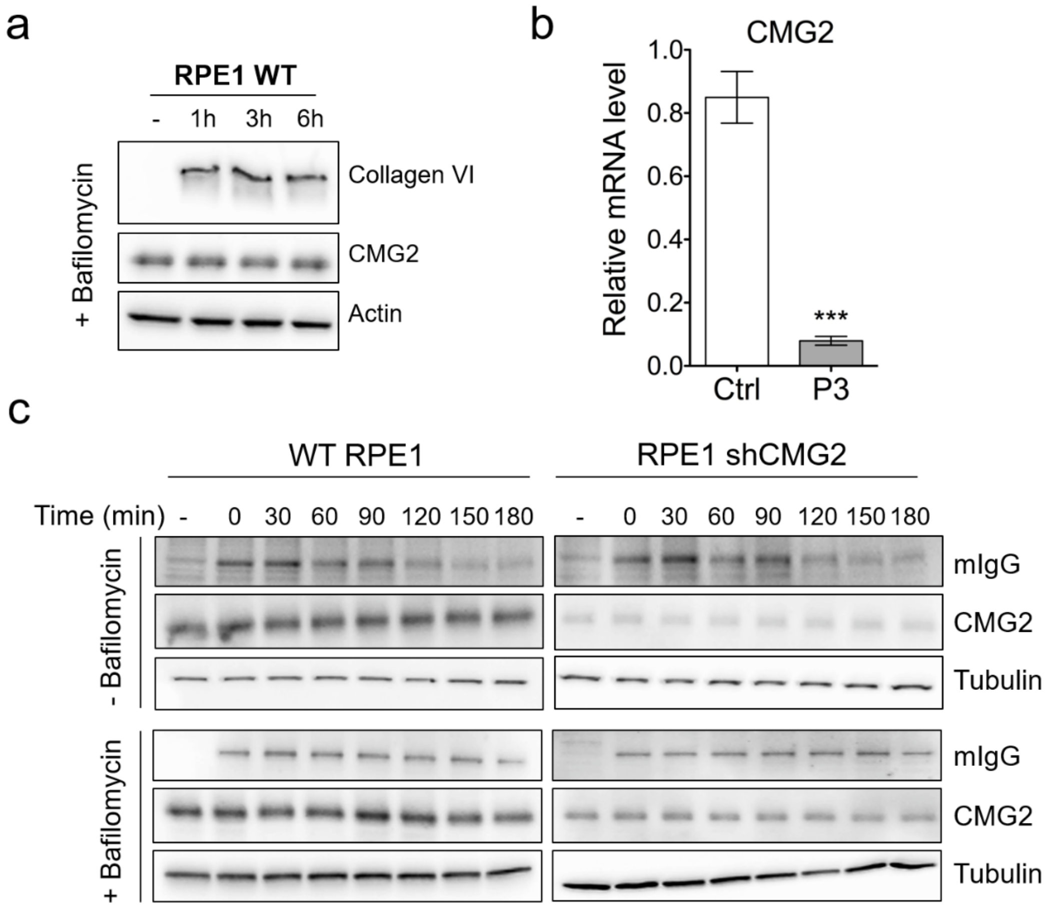

Supplementary Figure 6. (a) RPE1 cells were treated with Bafilomycin for 1 hour, blocked with 5% BSA, before addition of purified collagen VI tetramer at 1 µg/ml. Cells were harvested 1, 3 or 6 hours later. Collagen VI degradation was assessed by SDS-PAGE using 4-12% Bis-Tris gradient gels under non-reducing condition and western blotting for collagen VI, endogenous CMG2 and actin as a loading control. Collagen VI degradation was quantified by densitometric analysis and is shown in Fig. 6d. **(b)** Relative CMG2 mRNA level in control and P3 fibroblasts (Error bars represent s.e.m; n = 3; *, p < 0.05, two-tailed unpaired t-test compared to control). **(c)** RPE1 cells knock-down or not for CMG2 were incubated with mouse IgG and their degradation with or without bafilomycin was monitored for 8 hours and analyzed by SDS-PAGE using 4-12% Bis-Tris gradient gels under reducing condition and western blot against mouse IgG, CMG2 and tubulin as a loading control.

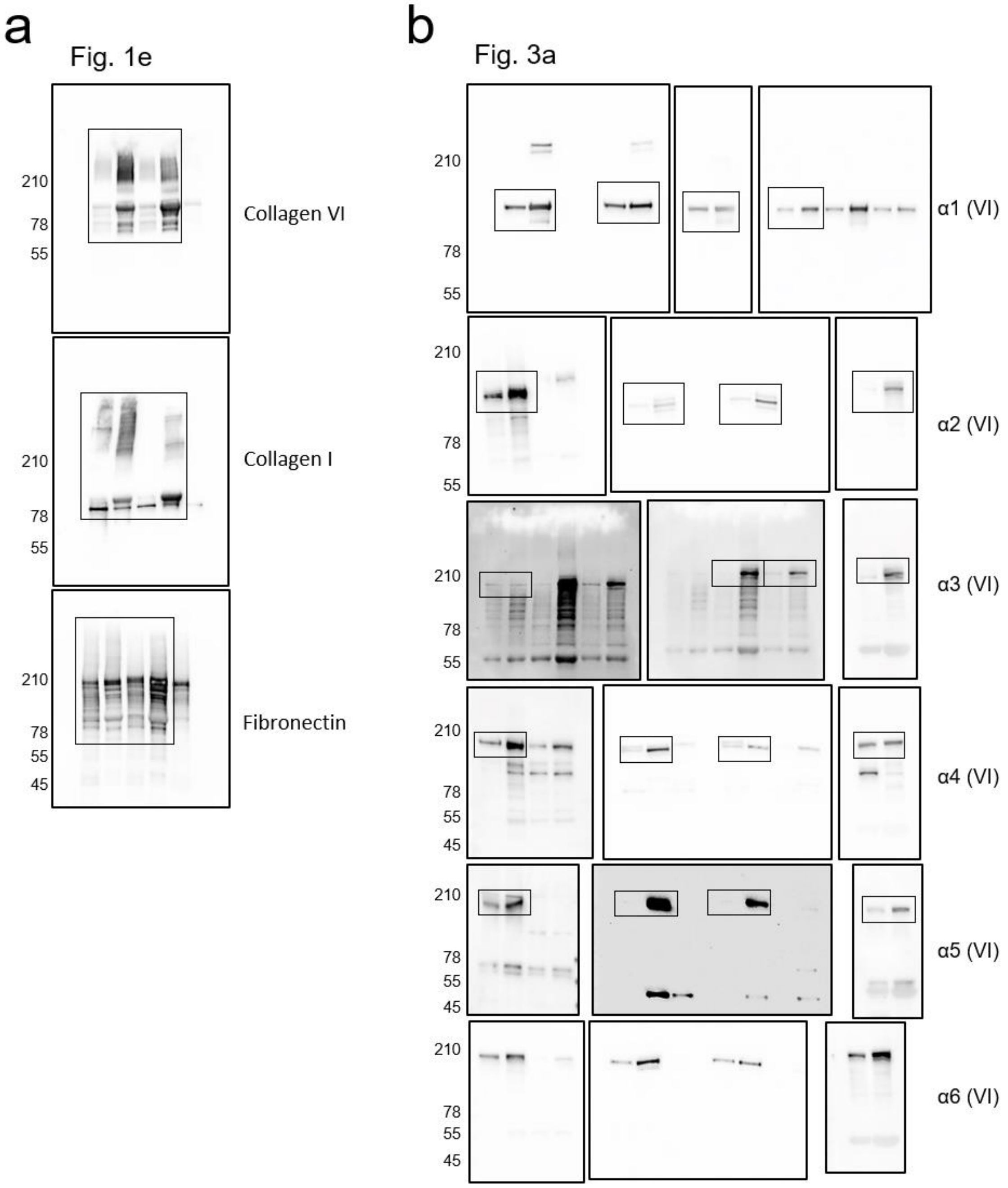

Supplementary Figure 7. Uncropped images of the western blots analysis of the main figures. The molecular weight is indicated at the left of the immunoblots (in kDa). The black rectangle indicates the part that was kept for the final figure. **(a)** Corresponding images to Fig. 1e. **(B)** Corresponding images from Fig. 3a.

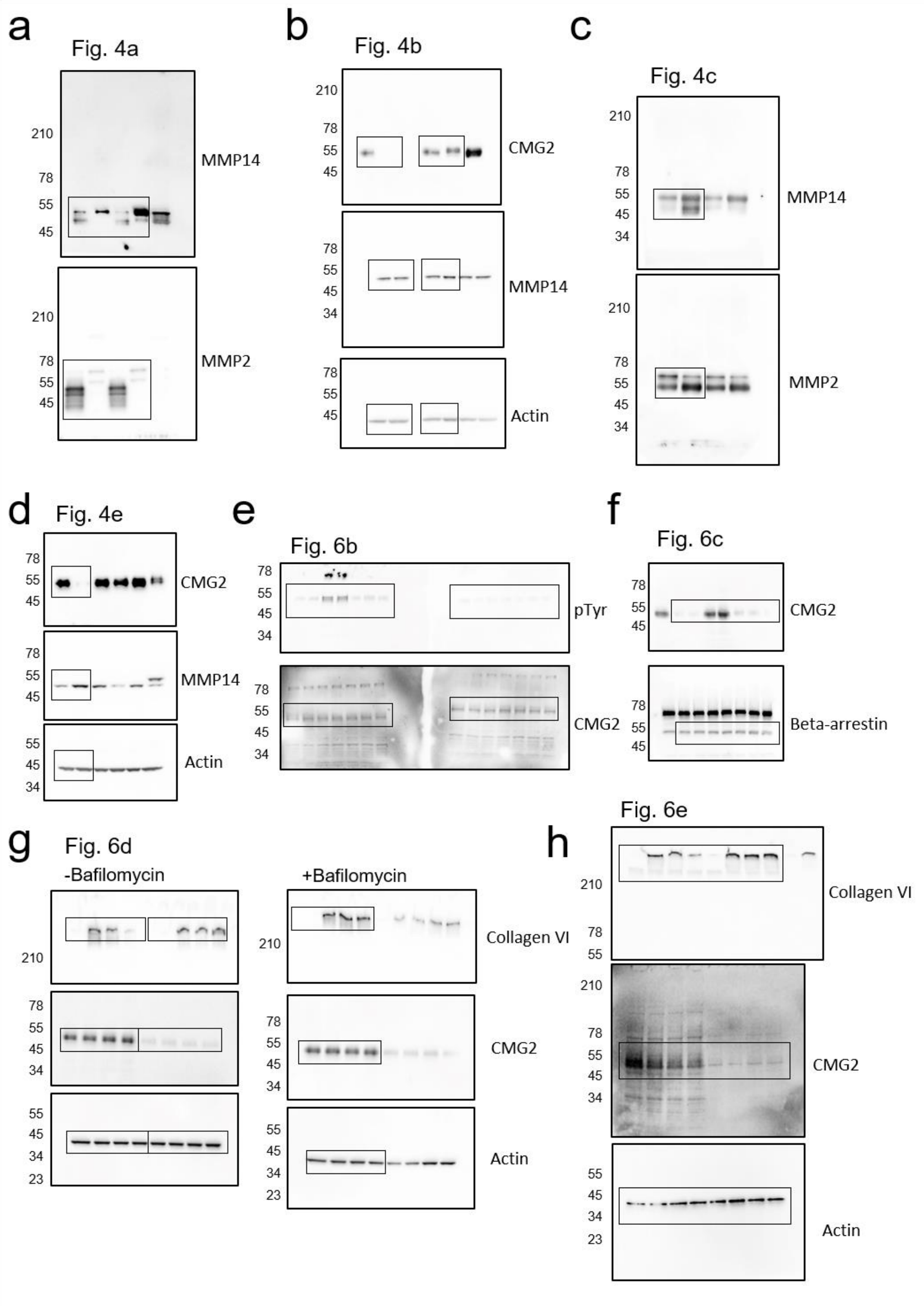

Supplementary Figure 8. Uncropped images of the western blots analysis of the main figures. The molecular weight is indicated at the left of the immunoblots (in kDa). The black rectangle indicates the part that was kept for the final figure. **(a)** Corresponding images to Fig. 4a. **(b)** Corresponding images from Fig. 4b. **(c)** Corresponding images to Fig. 4c. **(d)** Corresponding images to Fig. 4e. **(e)** Corresponding images to Fig. 6b. **(f)** Corresponding images to Fig. 6c. **(g)** Corresponding images to Fig. 6d. **(h)** Corresponding images to Fig. 6e.

Supplementary Note 1. The index patient is a seven year-old Austrian girl who presented with painful contractures of large and small joints from the first weeks of live. She was born as the first child of healthy, distantly related parents at term after an uneventful pregnancy. Parameters for length and weight were normal at birth. In early infancy, she ceased to thrive and developed progressive growth failure. At four months of age, papulous skin lesions appeared on nasolabial folds, nose, ears, and the perianal region and evolved into rigid subcutaneous nodular tumors. These demarcated nodules slowly expanded in number and size. At 15 months, gingival hypertrophy was noted for the first time and recurred quickly each time after resection. Surgical removal or reduction was also tried several times for the subcutaneous perianal and retro-auricular tumors which regrew over a time of months, or years. Histopathology revealed a delimited hypocellular accumulation of homogeneous eosinophilic hyaline material. At the age of two years, a diagnosis of HFS was suspected on basis of clinical and radiographic findings. Subsequent targeted Sanger sequencing analysis demonstrated compound heterozygous c. [1074delT];[1223T>C] mutations of the *ANTXR2* gene (Supplementary Figure 1a). While the c.1074delT, mutation was previously shown to cause a frame shift and premature stop (p.R359H*fs**50) which leads to a complete loss of the truncated CMG2 protein by proteasomal degradation[5], the c.1223T>C transition predicts a novel p.L408P missense change in the intracellular domain of CMG2.

Supplementary References

1. Lacy, D. B., Wigelsworth, D. J., Scobie, H. M., Young, J. A. T. & Collier, R. J. Crystal structure of the von Willebrand factor A domain of human capillary morphogenesis protein 2: An anthrax toxin receptor. *Proc. Natl. Acad. Sci.* **101,** 6367–6372 (2004).

2. Deuquet, J., Lausch, E., Superti-Furga, A. & van der Goot, F. G. The dark sides of capillary morphogenesis gene 2: The dark sides of capillary morphogenesis gene 2. *EMBO J.* **31,** 3–13 (2012).

3. Hakki, S. S. *et al.* Periodontal treatment of two siblings with juvenile hyaline fibromatosis. *J. Clin. Periodontol.* **32,** 1016–1021 (2005).

4. Hatamochi, A., Sasaki, T., Kawaguchi, T., Suzuki, H. & Yamazaki, S. A novel point mutation in the gene encoding capillary morphogenesis protein 2 in a Japanese patient with juvenile hyaline fibromatosis. *Br. J. Dermatol.* **157,** 1037–1039 (2007).

5. Deuquet, J. *et al.* Hyaline Fibromatosis Syndrome inducing mutations in the ectodomain of anthrax toxin receptor 2 can be rescued by proteasome inhibitors: Hyaline Fibromatosis Syndrome mutations. *EMBO Mol. Med.* **3,** 208–221 (2011).

6. Deuquet J, Lausch E, Guex N, Abrami L, Salvi S, Lakkaraju A, et al. Hyaline Fibromatosis Syndrome inducing mutations in the ectodomain of anthrax toxin receptor 2 can be rescued by proteasome inhibitors: Hyaline Fibromatosis Syndrome mutations. EMBO Mol Med. 2011 Apr;3(4):208–21.

Supplementary Table 1

List of most enriched or depleted proteins in HFS patient nodules compared to non-nodular tissue according to peptides detected by Nano-LC MS/MS.

Identified Proteins	Accession Number	Molecular Weight	Ear Non Nodular (peptides)	Ear Nodule (peptides)	Head Non Nodular (peptides)	Head Nodule (peptides)	FC Ear Nod/Nn	FC Head Nod/Nn
**Eukaryotic translation initiation factor 5A-1**	P63241	17 kDa	11	4	9	1	0.41	0.07
**Tubulin alpha-4A chain**	A8MUB1	48 kDa	5	1	6	1	0.10	0.08
**Caspase-14**	P31944	28 kDa	25	1	4	1	0.02	0.13
**14-3-3 protein sigma**	P31947	28 kDa	19	1	13	1	0.03	0.04
**Histone H2B type 1-O**	P23527	14 kDa	11	1	26	1	0.04	0.02
**Protein S100-A9**	P06702	13 kDa	490	1	9	1	0.00	0.05
**Calmodulin-like protein 5**	Q9NZT1	16 kDa	39	1	12	1	0.01	0.04
**Galectin-7**	P47929	15 kDa	24	1	25	1	0.02	0.02
**Junction plakoglobin**	F5GWP8	66 kDa	22	1	35	1	0.02	0.02
**Periostin**	B1ALD9	90 kDa	11	56	1	26	5.17	51.26
**Collagen alpha-2(VI)**	P12110	109 kDa	11	186	9	100	16.59	11.33
**Collagen alpha-1(VI)**	P12109	109 kDa	18	227	15	160	12.78	10.40
**Alpha-1-antitrypsin**	P01009	47 kDa	30	201	32	230	6.71	7.22
**Apolipoprotein A-IV**	P06727	45 kDa	1	11	1	6	10.13	6.76
**Collagen alpha-3(VI)**	E9PCV6	322 kDa	19	109	15	101	5.63	6.66
**Collagen alpha-3(VI)**	P12111	344 kDa	19	102	15	94	5.31	6.48
**Apolipoprotein A-I**	P02647	31 kDa	47	432	88	530	9.18	6.04
**Transforming growth factor-beta-induced protein ig-h3**	G8JLA8	75 kDa	11	204	8	48	18.86	6.03
**Hemopexin**	P02790	52 kDa	2	15	3	15	6.49	5.98

FC: fold change

Supplementary Table 2

Patient fibroblasts used in this study

Patient	Zygosity	DNA	Protein	Exon	Localization	Severity	Ref.
P1	Hom.	N.D.	D50N	1	Extracellular	ISH	2
P2	Hom.	c.1153G>C	p.G385R	14	Cytoplasmic	JHF	This study
P3	Hom.	c.789-790delT	Frameshift	9	Extracellular	ISH	3
P4	Hom.	c.1156G>T	p.V386F	14	Cytoplasmic	JHF	4
P5	Hom.	c.1074insC	Frameshift	13	Cytoplasmic	ISH	6
Control	Hom.	WT	WT	-	-	-	

Patient mutations were already described: P1[2], P2 (this study), P3[3], P4[4], P5[6]

Supplementary Table 3

qPCR primers used in this study

Human	Forward	Reverse
Col1a1	CCCGAGGCTCTGAAGGTC	GAGCACCATTGGCACCTTT
Col1a2	ACAAGGCATTCGTGGCGATA	ACCATGGTGACCAGCGATAC
Col4a1	TAGGCACAGGACCTTTGGGA	TGGGAAACCTTTTGGGCCTG
Col4a2	CCAGGTTTTAAAGGCAGCCG	TTTGCGCCCAGGTATCCTTT
Col6a1	TCTGCATAGACAAGAAGTGTCCA	GGTGTCAAAGTTGTGGCTGC
Col6a2	CCTGGTCGCTGAGAAGTTCA	CACGGACAGCTCTGTTTGG
Col6a3	AACATCGGCACTTGCCCTTA	CGCACCATTTTTGACATCTGC
TBP	GCCCGAAACGCCGAATATA	CGTGGCTCTCTTATCCTCATGA
B2 Microglobulin	TGCTCGCGCTACTCTCTCTTT	TCTGCTGGATGACGTGAGTAAAC
**Mice**	**Forward**	**Reverse**
Col1a1	GTTCAAGGTCCCCCAGGCCC	GGCTACCAGGTCCACCACGC
Col1a2	CTGGCGCCAAGGGTGCTACT	GCAGGACCAGGCTCACCAACA
Col4a1	CAGGGGCCTCCGGGAGAGAT	ACCTTGTGGACCCGGCAATCC
Col4a2	TGGGCCCCCAGGGGTACAAT	ACGCCTCTCGCTCCCACATC
Col6a1	TGCCCTGTGGATCTATTCTTCG	CTGTCTCTCAGGTTGTCAATG
Col6a2	CATCTCACCCCAGGAGCAGGAA	TACACGTTGACTGGGCAGTCGG
Col6a3	AACCCTCCACATACTGCTAATTC	TCGTTGTCACTGGCTTCATT
Col6a4	ATGACAAGTGCCGACCAGCC	ACTAGCCGCAAAGCCCCAAG
Col6a5	TGCTCTGTTGGTGGTGTCCC	TGCCCAGGTCTAGCATCCCA
Col6a6	TTCAGTGCACAGAGGGGCAG	GACAGCTGCCTTGGTCACGT
TGF-Beta1	CACCGGAGAGCCCTGGATA	TGTACAGCTGCCGCACACA
TNF	ACAGAAAGCATGATCCGCG	GCCCCCCATCTT TTGGG
MMP14	CCCGGGTACCCCAAGCACAT	ACCGGTAGTACTTATTGCCCCGGAA
MMP2	CTCTATGGGCCCTCCCCCGA	ACCACGGATCTGAGCGATGCC
MMP8	AAACGGAGTGAGAGGTGTGG	TCTGCCTGGGAACTTATTGG
MMP9	TGCGCCACCACAGCCAACTA	TACGGTCGCGTCCACTCGG
SMA	TCAGCGCCTCCAGTTCCT	AAAAAAAACCACGAGTAACAAATCAA
Col3a1	GATGAGGAGCCACTAGACTG	GCCATCAGGAAGCACAGG
LOX	TCTTCTGCTGCGTGACAACC	GAGAAACCAGCTTGGAACCAG
Beta Actin	CTAAGGCCAACCGTGAAAAGAT	CACAGCCTGGATGGCTACGT
Cox6a1	CTCTTCCACAACCCTCATGTGA	GAGGCCAGGTTCTCTTTACTCATC
